# Supplementary material for: Advancing high-throughput combinatorial aging studies of hybrid perovskite thin films via precise automated characterization methods and machine learning assisted analysis
Source: J Mater Chem A Mater. 2024 Feb 6;12(12):7025–35. doi: 10.1039/d3ta07274f (PMC10950304; doi:10.1039/d3ta07274f)
Supplement: TA-012-D3TA07274F-s001 [file TA-012-D3TA07274F-s001.pdf]

Electronic Supplementary Material (ESI) for Journal of Materials Chemistry A.
This journal is © The Royal Society of Chemistry 2024

Supporting Information for

Advancing High-Throughput Combinatorial Aging Studies of Hybrid Perovskite Thin-Films *via* Precise Automated Characterization Methods and Machine Learning Augmented Analysis

Alexander Wieczorek[a], Austin G. Kuba[b], Jan Sommerhäuser[a], Luis N. Caceres[a], Christian M. Wolff[b,*], Sebastian Siol[a,*]

[a]*Laboratory for Surface Science and Coating Technologies, Empa − Swiss Federal Laboratories for Materials Science and Technology, Switzerland.*

[b]*Institute of Electrical and Microengineering (IEM), Photovoltaic and Thin-Film Electronics Laboratory, EPFL − École Polytechnique Fédérale de Lausanne, Switzerland.*

*Corresponding authors: christian.wolff@epfl.ch, sebastian.siol@empa.ch

Table of Figures

Figure S 1. Schematic of the beam path and position of the beam splitter and collimators. The light intensity of the light source I_0 is attenuated through the sample transmission or reflection. 2

Figure S 2. a) Measured reflectance and transmittance of a combinatorial library, showing interference fringes around the bandgap of approx. 1.6 eV. b) Resulting reflection corrected transmittance. 2

Figure S 3. A) Calibrated spectral intensity of the LED light source used against the AM 1.5G spectrum. B) Spectral external quantum yield (EQY) of the photodiode used to set the light intensity of the LED light source. 2

Figure S 4. A.) Spatial homogeneity of the stress light across the library area. B.) Temporal stability of the stressing light. Following a slight intensity overshoot of up to 6%, an intensity plateau is reached after approx. 6 min which corresponds to the calibrated light intensity. The signal intensity at 580 nm was selected in both cases as the emission maximum of the used LED light source. 3

Figure S 5. False-colour thermal image of climate chamber with marked temperatures across the combinatorial grid, showing a standard deviation of <1 °C on perovskite thin-film library. An offset from the actual temperature of 85 °C resulted from the low emissivity of the samples on the substrate. 3

Figure S 6. 2Θ XRD measurements at shallower angles performed a) before and b) after the aging procedure. One sample exhibited a feature at approx. 9.8 °, indicative of residual unreacted MAI on the thin-film[1] which was however absent in the aged thin-films. Overconverted perovskite thin-films (*i.e.* those with overstochiometric amounts of A-site cations) can typically be detected at these shallower diffraction angles.[2,3] However, no similar feature could be detected at the angle previously reported for overconverted $MAPbI_3$ thin-films (blue marker).[1] 4

Figure S 7. XRD diffractogram of library samples measured within the inert-gas XRD Dome before (blue lines) and after (green lines) 17 h within the Dome. No significant change of the peaks was observed based on the differential spectrum (red dots). 4

Figure S 8. Overlayed mapping of the MHP/PbI_2 phase ratio on top of a photograph of the pristine library. Behind the patterned library on the transparent substrate, an aluminum foil can be seen. 5

Figure S 9. Absorption coefficient α vs Photon Energy for a thermally evaporated PbI_2 template and a resulting $MAPbI_3$ thin-film with a high conversion rate with band gaps of approx. 2.4 eV and 1.6 eV, respectively. 5

Figure S 10. Tauc plot analysis for an indirect allowed transition with varying φ. A shift of less than 0.2 eV can be observed in the extracted band gaps from samples with the highest and lowest φ values. 6

Figure S 11. Feature importance of the optimized XGBRegressor model. 6

Figure S 12. Regressor model applied on samples with high φ values of > 2.5. 7

Figure S 13. Distribution of MHP/PbI_2 phase ratio within the training and test data expressed as a histogram with an overlayed probability distribution function. Samples with a MHP/PbI2 phase ratio of φ > 2.5 constitute the minority, amounting for only 20% of the total training data. The distribution function exhibites the similar shape for both data sets, indicating a likewise represention of the biased distribution within both the training and test data. 7

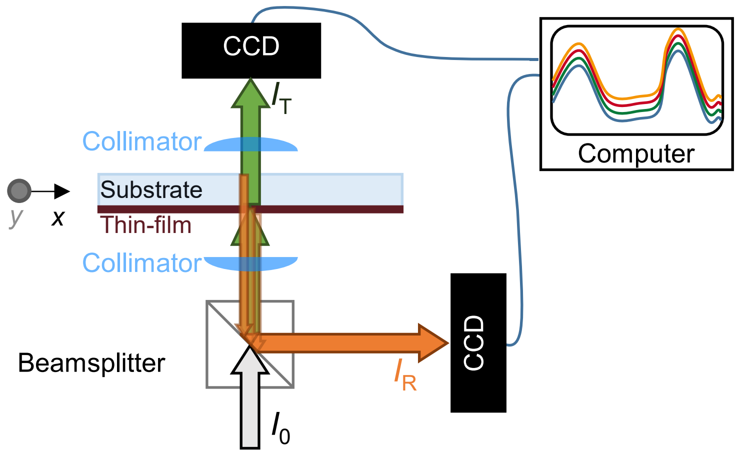

Figure S 1. Schematic of the beam path and position of the beam splitter and collimators. The light intensity of the light source I_0 is attenuated through the sample transmission or reflection.

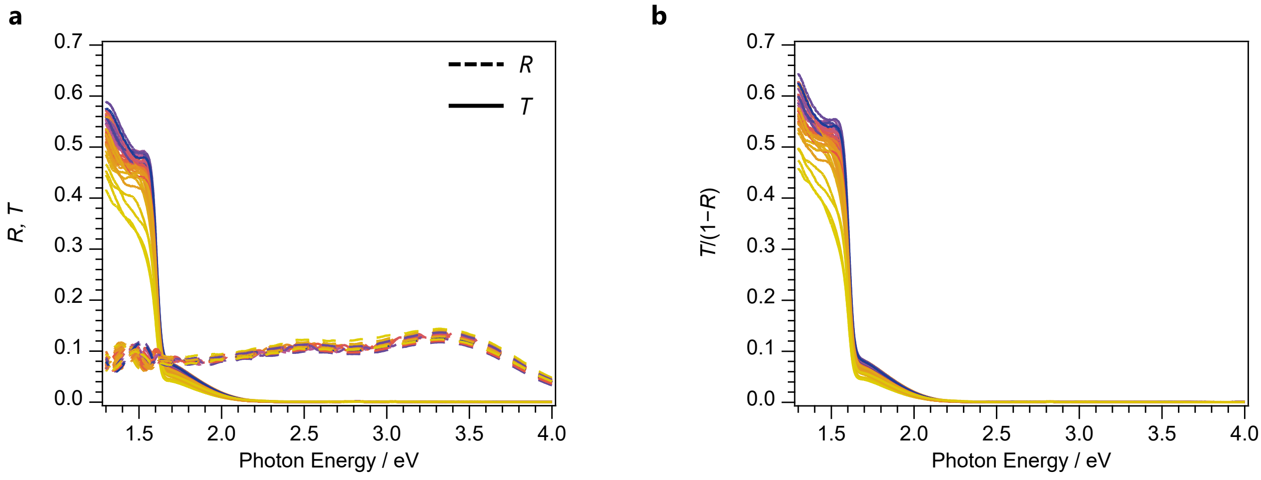

Figure S 2. a) Measured reflectance and transmittance of a combinatorial library, showing interference fringes around the bandgap of approx. 1.6 eV. b) Resulting reflection corrected transmittance.

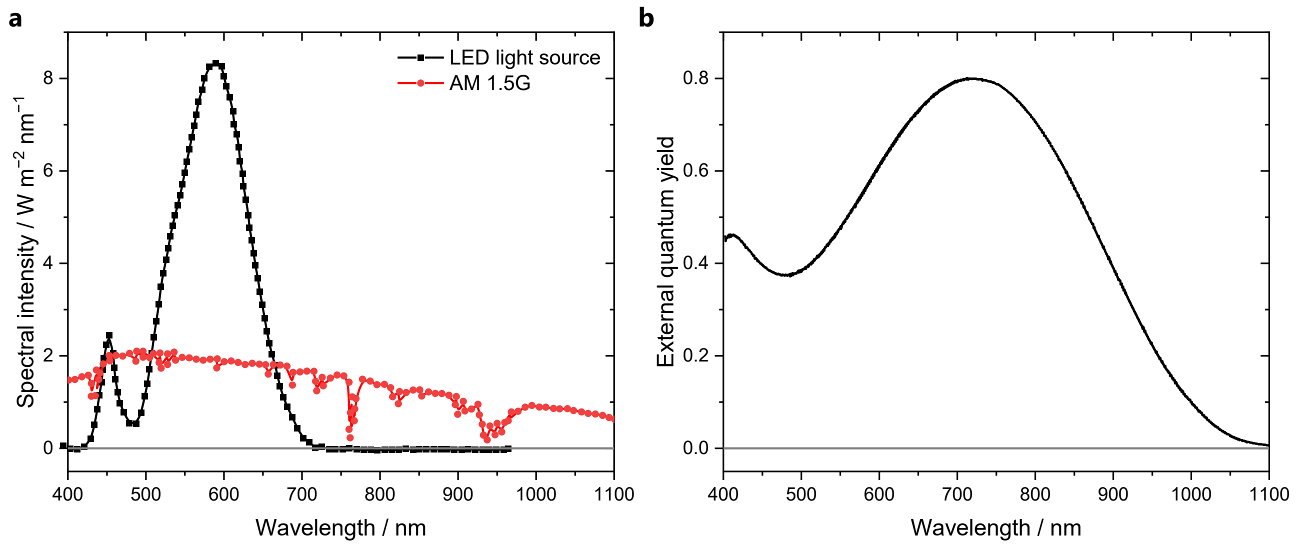

Figure S 3. A) Calibrated spectral intensity of the LED light source used against the AM 1.5G spectrum. B) Spectral external quantum yield (EQY) of the photodiode used to set the light intensity of the LED light source.

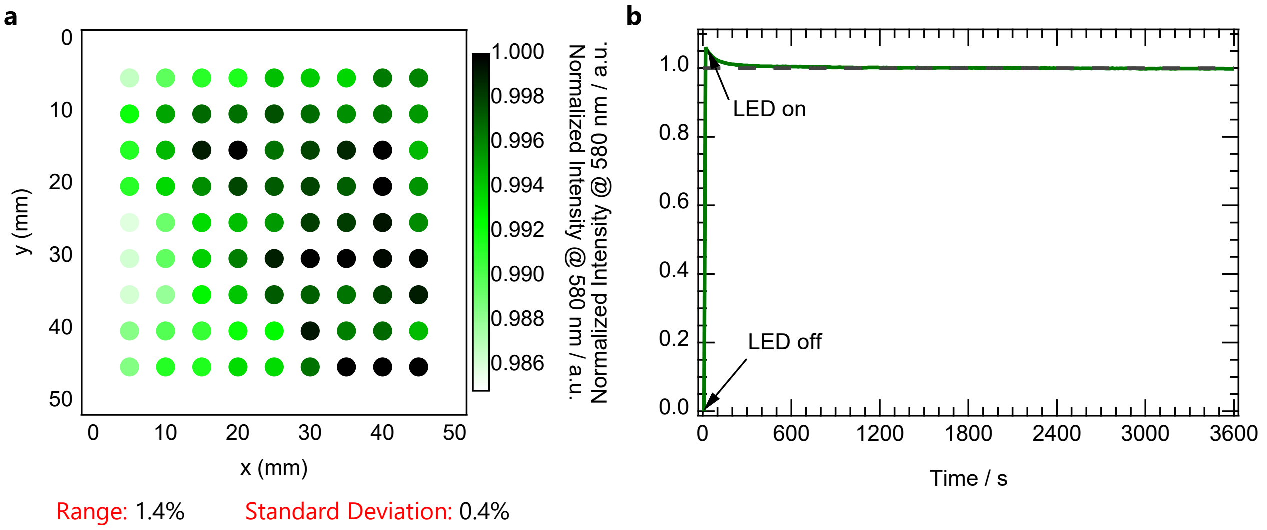

Figure S 4. A.) Spatial homogeneity of the stress light across the library area. B.) Temporal stability of the stressing light. Following a slight intensity overshoot of up to 6%, an intensity plateau is reached after approx. 6 min which corresponds to the calibrated light intensity. The signal intensity at 580 nm was selected in both cases as the emission maximum of the used LED light source.

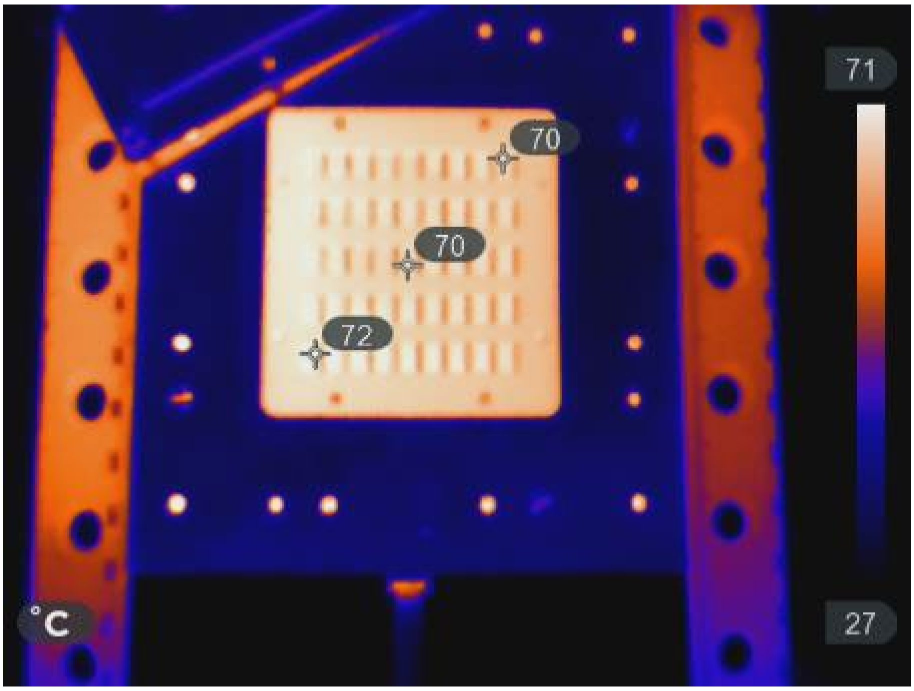

Figure S 5. False-colour thermal image of climate chamber with marked temperatures across the combinatorial grid, showing a standard deviation of <1 °C on perovskite thin-film library. An offset from the actual temperature of 85 °C resulted from the low emissivity of the samples on the substrate.

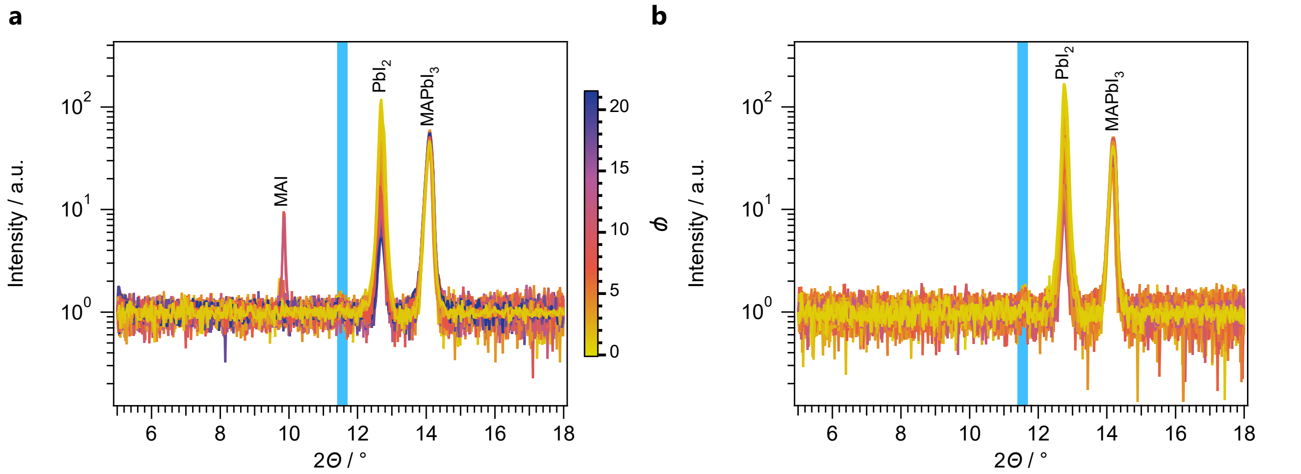

Figure S 6. 2Θ XRD measurements at shallower angles performed a) before and b) after the aging procedure. One sample exhibited a feature at approx. 9.8 °, indicative of residual unreacted MAI on the thin-film[1] which was however absent in the aged thin-films. Overconverted perovskite thin-films (*i.e.* those with overstochiometric amounts of A-site cations) can typically be detected at these shallower diffraction angles.[2,3] However, no similar feature could be detected at the angle previously reported for overconverted $MAPbI_3$ thin-films (blue marker).[1]

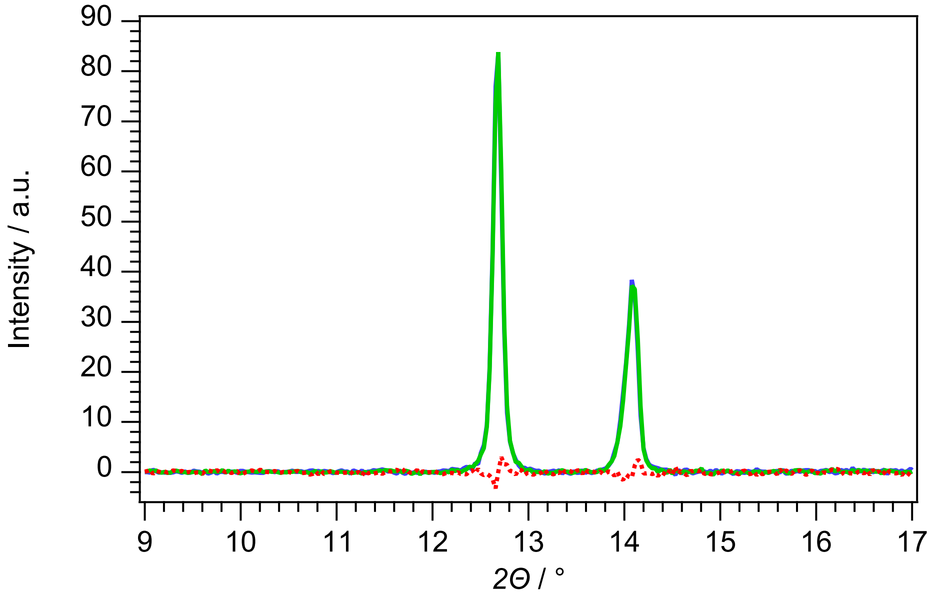

Figure S 7. XRD diffractogram of library samples measured within the inert-gas XRD Dome before (blue lines) and after (green lines) 17 h within the Dome. No significant change of the peaks was observed based on the differential spectrum (red dots).

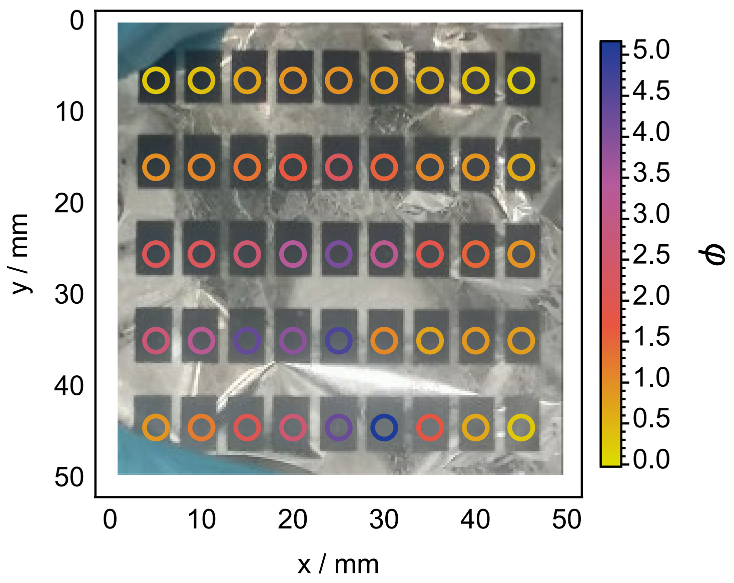

Figure S 8. Overlayed mapping of the MHP/PbI_2 phase ratio on top of a photograph of the pristine library. Behind the patterned library on the transparent substrate, an aluminum foil can be seen.

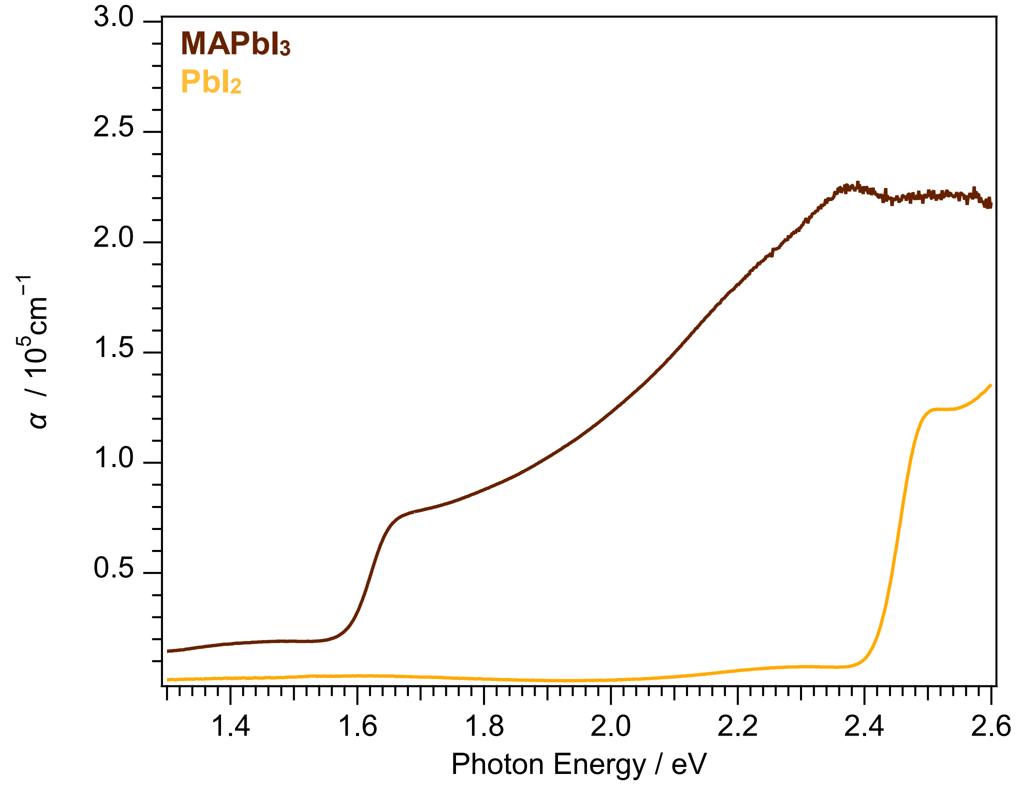

Figure S 9. Absorption coefficient α vs Photon Energy for a thermally evaporated PbI_2 template and a resulting $MAPbI_3$ thin-film with a high conversion rate with band gaps of approx. 2.4 eV and 1.6 eV, respectively.

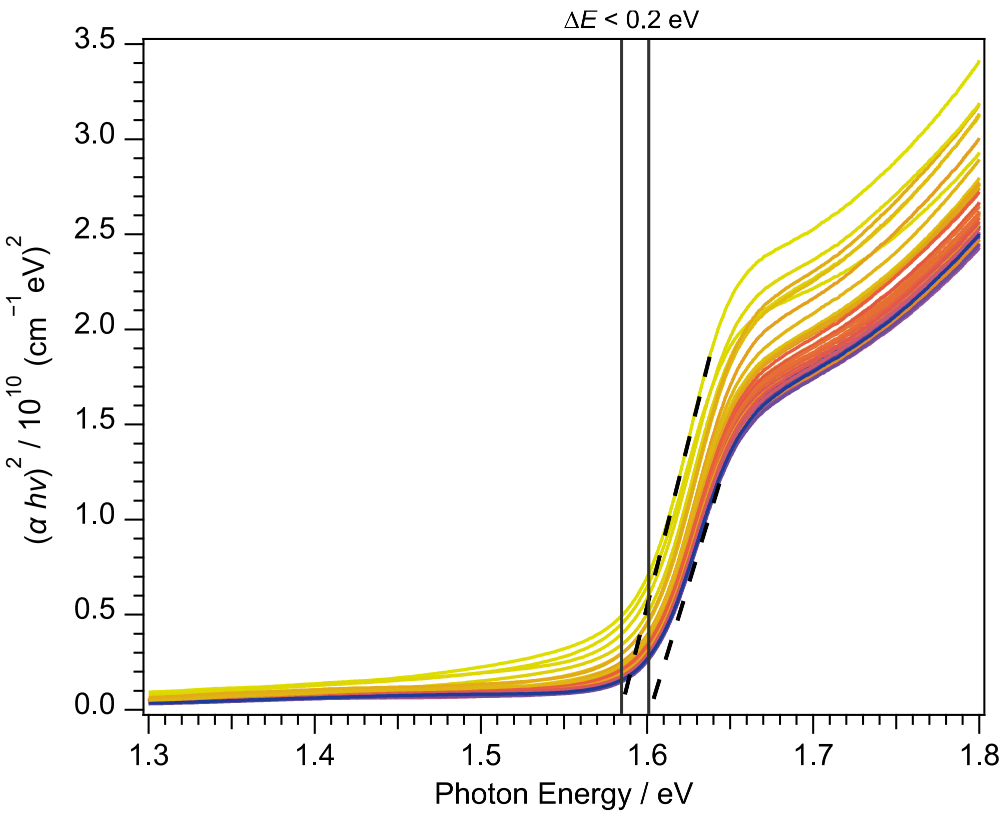

Figure S 10. Tauc plot analysis for an indirect allowed transition with varying φ. A shift of less than 0.2 eV can be observed in the extracted band gaps from samples with the highest and lowest φ values.

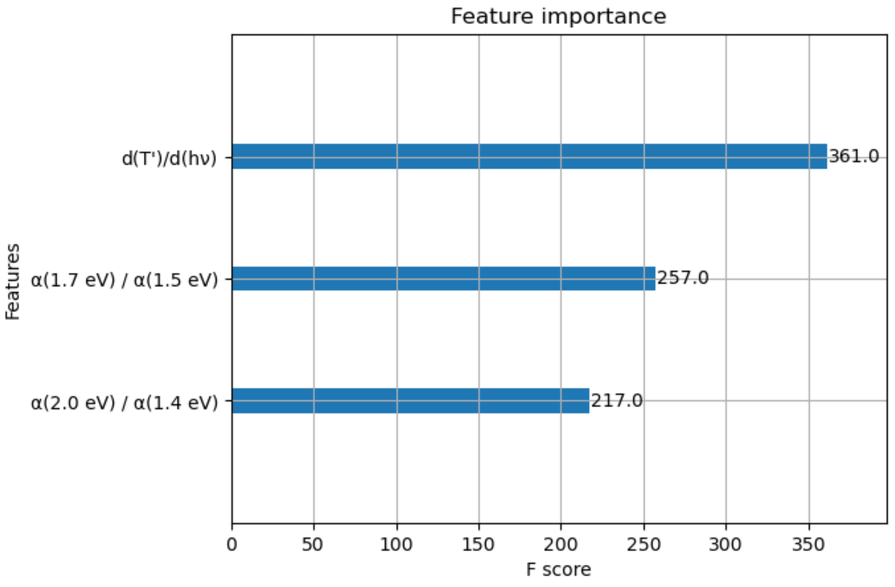

Figure S 11. Feature importance of the optimized XGBRegressor model.

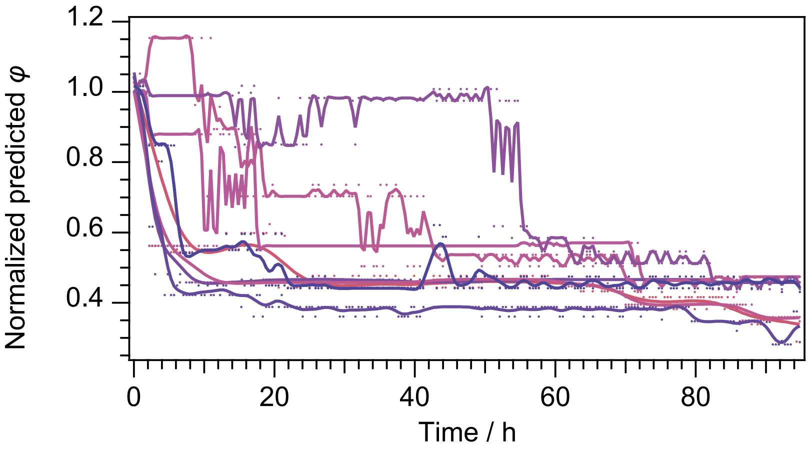

Figure S 12. Regressor model applied to samples with high φ values of > 2.5.

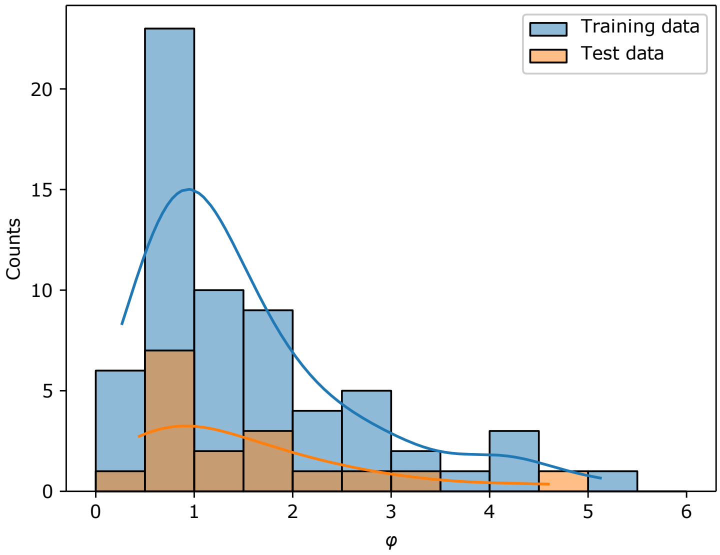

Figure S 13. Distribution of MHP/PbI_2 phase ratio within the training and test data expressed as a histogram with an overlayed probability distribution function. Samples with a MHP/PbI2 phase ratio of φ > 2.5 constitute the minority, amounting to only 20% of the total training data. The distribution function exhibits a similar shape for both data sets, indicating a likewise representation of the biased distribution within both the training and test data.

References

1 Z. Song, S. C. Watthage, A. B. Phillips, B. L. Tompkins, R. J. Ellingson and M. J. Heben, *Chem. Mater.*, 2015, **27**, 4612–4619.

2 T. Moser, K. Artuk, Y. Jiang, T. Feurer, E. Gilshtein, A. N. Tiwari and F. Fu, *J. Mater. Chem. A*, 2020, **8**, 21973–21982.

3 Q. Guesnay, C. J. McMonagle, D. Chernyshov, W. Zia, A. Wieczorek, S. Siol, M. Saliba, C. Ballif and C. M. Wolff, *ACS Photonics*, 2023, **10**, 3087–3094.
